# A New Perspective of *Pseudomonas*—Host Interactions: Distribution and Potential Ecological Functions of the Genus *Pseudomonas* within the Bark Beetle Holobiont

**DOI:** 10.3390/biology10020164

**Published:** 2021-02-19

**Authors:** Zaki Saati-Santamaría, Raúl Rivas, Miroslav Kolařik, Paula García-Fraile

**Affiliations:** 1 Microbiology and Genetics Department, University of Salamanca, 37007 Salamanca, Spain; raulrg@usal.es; 2 Spanish-Portuguese Institute for Agricultural Research (CIALE), Villamayor, 37185 Salamanca, Spain; 3 Associated Research Unit of Plant-Microorganism Interaction, USAL-CSIC (IRNASA), 37008 Salamanca, Spain; 4 Department of Botany, Faculty of Science, Charles University, 128 01 Prague, Czech Republic; mkolarik@biomed.cas.cz; 5 Institute of Microbiology of the Czech Academy of Sciences, Vídeňská 1083, 142 20 Prague, Czech Republic

**Keywords:** host–microbe interaction, forest pests, microbiota, *Dendroctonus*, insects microbiome, insect–microbe interactions, fungal antagonism, biocontrol, Scolytinae, symbionts

## Abstract

**Simple Summary:**

Microbes play essential roles in the health of animals and plants. Hence, the study of microbe–host interactions is of utmost importance to understand nature. In the present work, we aimed to understand the ecological distribution and functions of the bacterial genus *Pseudomonas* in bark beetles. These beetles are small insects that live under the bark of trees. Some bark beetle species cause mass attacks in woodlands, decimating tree populations worldwide. Thus, a better understanding of its associated microbes may aid in finding solutions for these forest pests. Our revision summarizes how members of the genus *Pseudomonas* are ubiquitous in all life stages of different bark beetle species. Moreover, we found that these bacteria may benefit these insects by providing them with nutrients, protecting them from tree chemical defenses and antagonizing entomopathogenic fungi.

**Abstract:**

Symbiosis between microbes and insects has been raised as a promising area for understanding biological implications of microbe–host interactions. Among them, the association between fungi and bark beetles has been generally recognized as essential for the bark beetle ecology. However, many works investigating bark beetle bacterial communities and their functions usually meet in a common finding: *Pseudomonas* is a broadly represented genus within this holobiont and it may provide beneficial roles to its host. Thus, we aimed to review available research on this microbe–host interaction and point out the probable relevance of *Pseudomonas* strains for these insects, in order to guide future research toward a deeper analysis of the importance of these bacteria for the beetle’s life cycle.

## 1. Introduction

Nature is maintained in a delicate balance, which is currently being greatly affected by the effects of climate change. Consequently, ecosystems are being reshaped, where some species are becoming extinct and others are invading new ecological niches and becoming pests [1]. Among these pests, bark beetles (*Coleoptera*: *Curculionidae*: *Scolytinae*) are responsible for significant damage to forests [2]. In general, these insects spend most of their lives—from egg to adult stages—under the bark of trees, only to emerge in search of new host plants during the reproductive stage, starting a new life cycle [3]. Ecologically, they fall into two separate groups: the wood boring ambrosia beetles, nutritionally dependent on cultivated ambrosia fungi and the phloem-feeding species, which feed mostly on nutrient-rich-bark and phloem [4]. These species of phloeophagous beetles feed on a narrow repertoire of tree species, usually involving one single genus [5]. In contrast, ambrosia beetles are broad generalists, attacking unrelated plant genera, and feed on a narrow set of associated ambrosia fungi. In addition, some beetles attack dead trees and others can cause severe damage to trees when high population levels are reached under certain environmental conditions [2].

The same as humans do, bark beetles contain their own microbiome. As found in other insects that interact with plant insects (for a revision see [6,7,8]), microbial associates cohabit within bark beetles, constituting an entity known as the “bark beetle holobiont” [9]. It has been reported how several of these microbes play an important role in the nutrition of their host [9,10,11], while some others are able to detoxify the environment, protecting the beetle from tree defense compounds [12] and many of them are known to protect the holobiont from pathogenic microbes [13].

The most conspicuous and well-studied forms of symbiosis between bark beetles and microbes are those established with externally living fungi such as ambrosia and ophiostomatoid species [4]. In contrast to ectosymbionts, little is known about the endosymbionts living in gut or hemolymph. Moreover, although several studies have focused on unveiling the paradigms of the fungus–beetle interaction over the decades [10,14], the bacteria–beetle interactions have only recently received increased attention [9].

A revision of the available literature describing bark beetles’ bacterial associates shows a wide distribution of bacteria belonging to the genus *Pseudomonas* (see next sections). *Pseudomonas* is a bacterial genus with great metabolic versatility, inhabiting many different environments and hosts [15,16,17,18]. *Pseudomonas* has evolved toward specific beneficial or pathogenic interactions, mainly with plants [19,20], humans [21], and insects [22,23,24].

Based on the increasing available literature involving bark beetle–bacterial interactions and the relatively large number of reports that identified *Pseudomonas* as part of the bacteriome of several bark beetles or proposed a relevant role of strains from this genus for their host, this review aims to provide an overview of the state-of-the-art in the association between *Pseudomonas* and bark beetles, reviewing the potential ecological roles of the microbe within the bark beetle holobiont. We also aim to highlight the existing gaps in the knowledge of this association, providing some clues to test unproven hypotheses on the reported functions of pseudomonads within their host. Finally, considering the worldwide impact of some bark beetle species as forest pests and the capability of some *Pseudomonas* species to act as insect pathogens, we conclude the review with a section in which the existing knowledge on the use of insect pathogens belonging to *Pseudomonas* species as biocontrol agents is also presented.

## 2. *Pseudomonas* Bacteria Are Broadly Associated to Bark Beetles

The bacteriome of the bark beetle holobiont has been characterized in many different culture-dependent studies. Diverse approaches have been developed to obtain bacterial isolates from different body-parts of the beetle such as oral secretions [25], gut [26], cuticle [27], frass [28], or even from the whole crushed insects [29]. In addition, some studies have isolated bacterial strains from the tree galleries where beetles feed [12,30]. Worldwide studies have been also performed to find constant associations in ambrosia beetles [31,32]. The most complex approach includes data from insect bodies of various life stages as well as analysis of the gallery content [33]. The revision of these and other studies points at a bacterial genus very frequently associated with bark beetles: the genus *Pseudomonas* (Table 1).

The bark beetle cultivable bacteriome has been studied mainly in individuals belonging to the genus *Dendroctonus* and there is a plethora of studies reporting the isolation of bacteria classified as *Pseudomonas* from this beetle. *Pseudomonas* strains have been found in *D. rhizophagus* collected from *Pinus arizonica* trees [26,39], oral secretions, surface, whole bodies, and galleries of *D. ponderosae* [41,50,54] and its tree host (*Pinus contorta*) phloem [12]; in gut, frass, surface, or whole bodies of *D. valens* in different life stages [28,30,40,43,48,52,53,59]; in the gut of *D. armandi* larvae [42]; in *D. micans* larvae and adults collected from *Picea orientalis* [46]; in *D. rufipennis* (collected from *Picea* sp.) and in *D. pondersoae* (sampled from *Pinus contorta*) oral secretions [25,46]; and in the gut of adults (but not larvae) of *D. frontalis* (obtained from galleries excavated in *Pinus taeda*) [58].

Apart from *Dendroctonus* beetles, Ceja-Navarro et al. [56] isolated *Pseudomonas* from the coffee berry borer (*Hypothenemus hampei*). Delalibera et al. [55] reported the presence of *Pseudomonas* strains in the guts of *Ips pini* pupae and adults. Sevim et al. [60] isolated *Pseudomonas* from crushed adults of *I. sexdentatus* living on *Picea orientalis* and Fabryová et al. [11] from adult specimens of *Cryphalus piceae* (*Abies alba*) and *Ips typographus* (*Picea abies*). Additionally, *Pseudomonas* bacteria have been isolated from *Pityogenes bidentatus*, *Ips*, *Hypothenemus*, and *Scolytodes* beetles [29,56,57] and experiments carried out in our laboratory showed the presence of *Pseudomonas* in *I. typographus*, *I. cembrae*, and in *Xylocleptes bispinus* (unpublished data).

Apart from phloeophagous bark beetles, *Pseudomonas* spp. have been cultivated from adults or larvae of ambrosia beetles such as *Xyleborus affinis*, *X. dispar*, and *Xylosandrus germanus* collected on hazelnut trees (*Corylus avellana*) [47,51,61].

Most of the previous ecological studies approach the analysis of the beetles’ microbiota through a single gene sequence taxonomic approach. However, broader polyphasic approaches have been applied in a few studies, which led to the discovery of novel species within the genus *Pseudomonas* such as *P. coleopterorum*, which was first isolated from *Hylesinus fraxini* [49], *P. typographi* discovered from *Ips typographi* [62], and *P. bohemica*, cultivated from *I. acuminatus* beetles [29].

Apart from the above-mentioned studies based on the isolation of the beetle bacterial associates, some other analysis of the microbial diversity associated with bark beetles based on culture-independent methods have detected the presence of *Pseudomonas* in the bark beetle holobiont (Table 1). Culture-independent approaches are based on massive sequencing of target genes (i.e., 16S rRNA gene, in the case of bacterial diversity studies) or on the shotgun sequencing of the whole DNA from the samples [33,63,64,65]. The major advantage of this latter approach is that it allows the inference of the functions linked to the microbiota [66] by comparison of the sequenced genes with databases of gene families and reference genomes.

Shotgun metagenomics in *D. ponderosae* beetles and galleries from different tree hosts—*Pinus contorta* (lodgepole pine) and *Pinus contorta*-*Pinus banksiana* (Hybrid pine)—and different locations showed *Pseudomonas* sequences being up to 60% of the total obtained bacterial sequences [38].

Aylward et al. [36] carried out a multi-platform analysis of different insects by combining 16S rRNA meta-barcoding and shotgun metagenome sequencing over larvae, adults, and galleries. They found that *Pseudomonas* sequences were predominant, not only in southern pine beetles and mountain pine beetles (60–95% of the sequences belongs to the family *Pseudomonadaceae*), but also in ambrosia beetles, fungus-growing ants, and termites. They also studied the phylogeny of reconstructed *Pseudomonas* genome bins, with average nucleotide identity (ANI) values up to 98% shared among pseudomonads of different origins, suggesting a functional convergence toward this microbe–host interaction.

Based on 16S rRNA amplicon sequencing analyses, *Pseudomonas* bacteria have been encountered in several *Dendroctonus* species (from different tree hosts): *D. frontalis* (*Pinus taeda*) [58], *D. adjunctus* (*Pinus hartwegii*), *D. approximatus* (*Pinus teocote*), *D. jeffreyi* (*Pinus jeffreyi*), *D. mesoamericanus* (*Pinus teocote*), *D. mexicanus* (*Pinus patula*), *D. parallelocollis* (*Pinus hartwegii*), *D. ponderosae* (*Pinus strobiformis*), *D. pseudotsugae* (*Pseudotsuga menziesii var. glauca*), *D. rhizophagus* (*Pinus arizonica*), *D. valens* (*Pinus leiophylla*), and *D. vitei* (*Pinus pseudostrobus)* [32], larvae, pupae, teneral adults, and pre-emerged and emerged adults of *D. rhizophagus (Pinus arizonica*) [34], crushed adults and larvae of *D. punctatus*, *D. micans* and *D. valens* [59], and cuticle and galleries of *D. simplex* (*Larix* × *Eurolepis*) [27].

Recently, Xu et al. [35] suggested that bacteria from the genus *Pseudomonas* are relevant members of the *core* microbiome in *D. valens*, finding that nearly 50% of the sequences obtained from larvae and adults belonged to this bacterial genus. Interestingly, these authors identified *P. bohemica* as one of the most abundant OTUs, this being a species first isolated from another bark beetle, *Ips acuminatus* [29]. Similarly, Mason et al. [30] found a higher proportion of pseudomonads in the galleries made by this beetle in *Pinus resinosa* than in the intact tree phloem.

Ceja-Navarro and colleagues [56], also using 16S rRNA amplicon sequencing, found that the genus *Pseudomonas* was prevalent in the *core* microbiome of *Hypothenemus hampei* isolated from different coffee trees worldwide (*Coffea arabica*, *C. arabica* var limaní, and *C. congensis* × *C. canephora*). However, using the same method and marker, *Pseudomonas* was found to be inconsistently associated with adults and eggs of the ambrosia beetle *Xyleborus affinis* [33] and a similar study over a most extended set of artificially reared ambrosia beetles (X*yleborus affinis*, *X. bispinatus*, *and X. volvulus*) showed that *Pseudomonas* is a rather uncommon symbiont, whose abundance is highly affected by the cultivation media used for the in vitro rearing [37].

Overall, considering studies based in both culture-dependent and independent methods, *Pseudomonas* bacteria have been encountered in 35 different bark beetle species from 11 genera (*Anisandrus*, *Dendroctonus*, *Ips*, *Xyleborinus*, *Hylesinus*, *Xyleborus*, *Xylocleptes*, *Pityogenes*, *Cryphalus*, *Hypothenemus*, and *Scolytodes*), which had been collected from 24 different tree species from nine different genera (*Pinus*, *Pseudotsuga*, *Coffea*, *Cecropia*, *Corylus*, *Larix*, *Fraxinus*, *Abies*, and *Picea*). Several of the reported studies did not separate different beetle tissues; however, based on the works in which a dissection of different beetle body-sites has been performed, we can conclude that bacteria belonging to *Pseudomonas* are associated with the bark beetles’ guts, cuticle, and oral secretions.

It is important to say that the above-mentioned culture independent analyses of the bark beetle bacteriome are based on short sequences of 16S rRNA amplicon fragments. These sequences do not allow a robust classification of the sequences at species level [67]. To obtain the most accurate identification at species level, there are nowadays massive parallel sequencing platforms with the potential to obtain nearly complete 16S rRNA gene sequences (~1500 bp), which allow a better identification of the microbiome at species level [35,37,68]. Moreover, amplicons of *Pseudomonas* housekeeping genes such as *rpoB*, *rpoD*, and *gyrB* [49] can be obtained for a more accurate identification at species level of those bacterial strains belonging to *Pseudomonas*. A more reliable identification at species level of the *Pseudomonas* associated with bark beetles will allow the analysis of a potential coevolution between *Pseudomonas* and *Scolytidae*.

In any case, based on all of the above-mentioned studies, we can conclude that bacteria from the genus *Pseudomonas* are widespread over bark beetles, being possibly part of the stable microbiome of these insects. Thus, it is possible that the existence of a co-evolution between bacteria from the genus *Pseudomonas* and bark beetles, in which the insects might have acquired abilities to survive and compete in their niches due to the bacterial metabolism and, similarly, the bacteria, might have gained fitness from their association with the host. However, further research should be made to prove an existing stable association between bark beetle species and *Pseudomonas*, in order to distinguish true coevolved associations from serendipitous, transient ones. For instance, sampling a beetle species along different locations and environmental conditions as well as life cycle stages would allow us to determine if certain *Pseudomonas* species belong to the core microbiome of the beetle species, and therefore to support the coevolution between the microbe and its host.

## 3. Role of *Pseudomonas* in the Nutrition of Bark Beetles

Bark beetles feed under the bark of their host trees, specifically on their phloem and/or their xylem [69,70]. The other dominant live strategy is that of ambrosia beetles, which nutritionally depend on cultivated ambrosia fungi [71]. Since the ingested plant tissues are constituted by complex molecules, insects are unable to easily degrade them [39]. However, they may get help: as a result of their enzymatic machinery, microbes present in the bark beetle holobiont can hydrolyze these complex tree polymers into more simple sugars, which can then be more available as nutrients to the insect host [9,11,39]. The most abundant polymers in the tree tissues, which the beetles feed on, are cellulose, xylan, and starch [11]. These polymers can be degraded by microbial enzymes: cellulose is degraded by cellulolytic enzymes called cellulases [49], xylan, the main component of hemicelluloses [72], by xylanases, and starch, another relevant polymer of plants, which is stored in granules, is digested by amylases [73,74].

**Figure 1:**
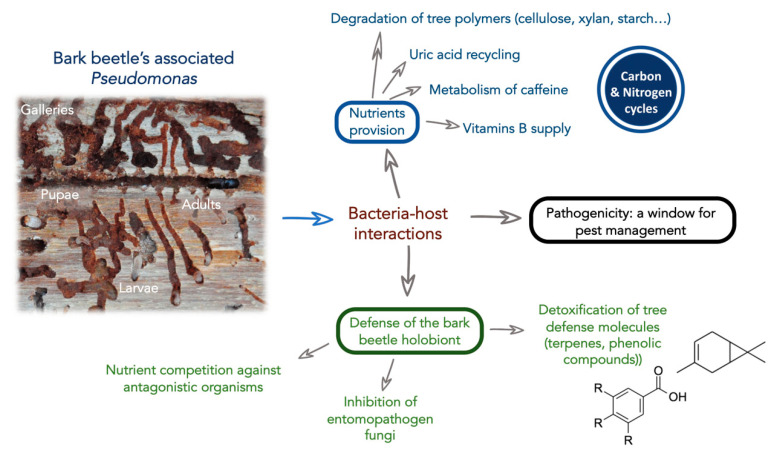
Scheme representing overall potential functions *Pseudomonas* in the bark beetle holobiont.

In this sense, some *Pseudomonas* strains isolated from bark beetles have been suggested to play a key role in the nutrition of its host (Table 1, Figure 1). For instance, *P. coleopterorum*, associated with *Hylesinus fraxini*, and *Pseudomonas* spp., isolated from the gut of *D. armandi*, have been characterized for their in vitro cellulolytic activity [42,49]. Additionally, in a broad study including many bacterial isolates from *C. piceae* and *I. typographus*, strains from the species *Pseudomonas arsenicoxydans* and *Pseudomonas trivialis* were able to degrade cellulose, xylan, and starch. These activities were detected by hydrolysis halos in Petri dishes containing these compounds. In a similar research, *Pseudomonas* strains closely related to *P. putida* and *P. azotoformans* species, isolated from larvae and adults’ guts from *D. rhizophagus*, were proven to have lipolytic, amylolytic, esterase, cellulolytic, and xylanolytic activities in vitro [39]. All these activities are related to the degradation of commonly available organic nutrients in the host tree of these beetles.

In addition to the degradation of complex plant polymers, *Pseudomonas* bacteria isolated from *Hypothenemus hampei*—beetles inhabiting coffee trees—can use caffeine as a sole source of carbon (C) and nitrogen (N) when grown in vitro. Indeed, when a *Pseudomonas* strain is inoculated into antibiotic-treated beetles, previously proven to be unable to metabolize caffeine, the ability to digest this molecule is restored, likely because of the ability of this bacterium to produce caffeine demethylase enzymes [56].

Furthermore, in vitro tests proved that *Pseudomonas* from the guts of *D. valens* can use uric acid as a sole source of energy, C, and N [40]. Since bark beetles excrete uric acid as a waste compound, this phenomenon may lead to its recycling and the authors suggest a contribution of these bacteria to the insect N balance, since its niche within the tree host is limited in N [40]. Beetles could assimilate recycled ammonium through the enzyme glutamine synthetase, combining ammonium and glutamate to form glutamine. However, the use of bacteria-mediated ammonium supply for the insect host has never been tested. As García-Fraile already pointed out [9], in vivo assays using gnotobiotic beetles inoculated with the *Pseudomonas* strains and uric acid labelled with the ^15^N stable isotope could allow for proof that the incorporation of ^15^N in the amino acids of the beetle is due to the bacterial capability to recycle uric acid.

Apart from C and N supply, another possible contribution to the bark beetle nutrition is the synthesis of vitamins. Many *Pseudomonas* strains have been characterized because of their ability to produce B-group vitamins [75,76,77,78,79]. The putative ability to produce B group vitamins and amino acids essential for insects was predicted in *Pseudomonas* associates of *X. affinis* [33]. In that study, the bacterial metabolic functions were predicted based on 16S rRNA sequences using the database of reference genomes. Thus, although to the best of our knowledge, the literature lacks studies directly reporting the relevance of these metabolic capabilities to beetle nutrition, the contribution of *Pseudomonas* to its host nutrition through the supply of vitamins and essential amino acids could be hypothesized, although rigorous in vivo tests to prove this hypothesis should be performed.

Most bark beetles are usually associated with *Ophiostomatales* fungi in a symbiotic relationship in which the beetle feeds on the fungi [44]. Interestingly, Adams and collaborators [50] showed how *Pseudomonas* spp. are able to both inhibit or promote the growth of different bark beetle fungal symbionts, depending on the fungal species and/or the presence of α-pinene: *Pseudomonas* spp. decreases the growth of *Ophiostoma montium*, but increases the growth of *Grosmannia clavigera* and *Leptographium procerum* when no α-pinene is present in the assay; notwithstanding, the bacterium increases the growth of all three fungi including also *O. montium*, plus *Ophiostoma ips*, when the bacterial treatment is combined with α-pinene.

All capabilities of bark beetle pseudomonads associates could be implicated in the promotion of beetle nutrition. Nonetheless, whether *Pseudomonas* strains of bark beetles play these roles in nutrition lacks rigorous research. These studies might include in vivo tests using gnotobiotic beetles inoculated with the *Pseudomonas* strains predicted to aid in nutrition and *knock-out* mutants in the metabolic pathways potentially implicated in the benefit to compare the beetle’s fitness in both cases.

## 4. Role of *Pseudomonas* in the Detoxification of the Bark Beetle Environment and in Pheromone Production

Trees are not passive to the massive attacks of bark beetles. They have many defenses including the production of volatile molecules such as phenolic compounds and terpenes, which are toxic to the beetles [9]. Nevertheless, these insects have some level of tolerance to these compounds [80]. Indeed, at low concentrations, these molecules are detected from adult beetles and help them to locate weakened host trees [9]. In addition, bark beetles produce pheromone molecules as a by-product of the degradation of host monoterpenes [81]. Several research studies have suggested that some bacteria associated with these insects are involved in the metabolization of tree defense compounds, which seems to result in the detoxication of the bark beetle’s environment [9].

It has been demonstrated that *Pseudomonas* inhabiting in the gut and frass of *D. valens* are able to metabolize the terpenes α-pinene and *cis-*verbenol and that *cis-*verbenol is converted to verbenone, a pheromone compound for bark beetles [43,82]. Although these biological activities were mainly demonstrated by in vitro tests [43,82], they are also supported by targeted metabolomic experiments carried out with *D. valens* guts with a large abundance of *Pseudomonas* in comparison to antibiotic-treated guts (guts without microbial associates) [41].

Similarly, *Pseudomonas* isolated from *D. ponderosae* decreased concentrations of the tree defense compounds 3-carene, (-)-α-pinene, (+)-α-pinene, and (-)-β-pinene in liquid cultures [12]. In addition, a *Pseudomonas* strain isolated from the red turpentine beetle (*D. valens*) was proven to grow in the presence of 1–5% of the monoterpenes α-pinene, myrcene, and 3-carene, and with a reduced growth in the presence of limonene and β-pinene [48].

Furthermore, this ability to degrade terpenes has also been studied through a shotgun metagenome analyses. Adams and colleagues [38] obtained samples of *D. ponderosae* adults, galleries, and unattacked tree phloem from different sites and compared their metagenome sequences. They found that samples associated with beetles were enriched in functions related with limonene and pinene degradation as well as genes belonging to the *dit* gene cluster, a well-characterized machinery involved in the degradation of diterpenes [83]. Indeed, most of these genes were taxonomically classified to *Pseudomonas* bacteria.

Based on all these studies, *Pseudomonas* seems to be broadly related with the chemical detoxification of the beetle’s environment, facilitating the survival of their insect host inside the tree (Table 1, Figure 1).

## 5. Role of *Pseudomonas* in the Defense of the Bark Beetle Holobiont against Pathogens

Tree chemical compounds are not the only dangers that bark beetles face; although these insects establish beneficial interactions with some fungi [84,85], some others are their antagonists, either causing disease or competing for nutrients [9,10]. In these detrimental associations, bacteria can serve as a defense for the beetle because of their ability to inhibit some of these fungal enemies [9,13,47,86].

Among these protective bacteria, *Pseudomonas* are important allies. Some volatile compounds produced by *Pseudomonas* isolated from *D. valens* decrease the in vitro growth of the antagonistic fungi *Ophiostoma minus* and *Leptographium procerum* [52,53]. Moreover, these *Pseudomonas* strains also have the ability to increase the growth of larvae inoculated with *O. minus* or *L. procerum* [52,53]. Similar findings were demonstrated by Zhou and collaborators [28]: apart from the alleviation of these fungal antagonistic effects, the authors showed how the consumption of D-pinitol and D-glucose by *O. minus* seemed decreased in the presence of pseudomonads, while, surprisingly, they forced *L. procerum* to consume D-pinitol prior to the consumption of D-glucose, which is hypothesized to remain available for the beetle.

Additionally, other fungi can also be influenced by the presence of *Pseudomonas*: inoculation of these bacteria into *D. ponderosae* larvae reduced the antagonistic effects of *Aspergillus* and *Trichoderma*, which led to more larvae and longer galleries. This effect was also observed in the new beetle tree host, jak pine (*Pinus banksiana*), recently accessed due to warming climate; this suggests that climate-driven expansion of *D. ponderosae* will not be significantly limited by the requirements of these beneficial bacterial associates [54].

Additionally, *Pseudomonas* strains isolated from *Ips acuminatus*, *Pityogenes bidentatus*, and *Cryphalus piceae* inhibit the growth of *Aspergillus* sp. in vitro [29]. Finally, *Pseudomonas chlororaphis*, a species associated with bark beetles [61] together with other pseudomonads, are known for their nematocidal activity [86,87], which could suggest a possible involvement of these bacteria in the control of bark beetle pathogenic nematodes.

Still on this subject, an in vitro test demonstrated that a *Pseudomonas viridiflava* strain isolated from oral secretions of *D. rufipennis* decreased the growth of four gallery-invasive fungal species: *Leptographium abietinum*, *Aspergillus fumigatus*, *Aspergillus nomius*, and *Trichoderma harzianum* [25]. These findings were obtained while the authors investigated the antifungal activity of the bark beetles’ oral secretions against these fungi, demonstrating that the antifungal activity appeared in non-filtered oral secretions and it disappeared when filter-sterilization was applied. Thus, bacteria from oral secretions, and among them, the species *Pseudomonas viridiflava*, seem to protect the beetle against these pathogenic fungi.

All these data suggest a role of *Pseudomonas* in the bark beetles’ pathogen exclusion (Table 1). This protective role of *Pseudomonas* and other bark beetle associated bacteria might in part explain the fact that entomopathogenic fungi, when applied as biocontrol agents for bark beetle forest pests, have high differences in effectiveness among distinct bark beetle species [88]. The application of this knowledge in integrated biocontrol strategies, targeting not only the beetle, but also its potential protective symbionts, may lead to an alleviation in the variations of susceptibility of bark beetles to entomopathogenic fungi.

## 6. Pathogenic *Pseudomonas:* Potential as Biocontrol Agents for Bark Beetle Pests

Along the previous sections of this review, we bring the idea of the potential benefiting roles of bacteria from the genus *Pseudomonas* for the bark beetle holobiont. However, with more than 200 validated species, *Pseudomonas* is a very broad bacterial genus with very diverse metabolic capabilities and lifestyles, and there is a plethora of studies in which the ability of *Pseudomonas* strains to become insect entomopathogens has been reported [22,89,90].

Among them, strains of the *P. fluorescens* group of species are well known insect pathogens. For instance, a study performed by Sevim et al. [60] demonstrated that the inoculation of a strain of *P. fluorescens* was capable of increasing the mortality of *Ips sexdentatus* larvae up to 73%. This strain with insecticidal activity was isolated from *I. sexdentatus* with disease symptoms. Therefore, this bacterium provides an opportunity to be used as a biocontrol agent against this forest pest.

Although existing literature on this research topic is scarce, the above-mentioned studies show the potential of bacterial strains as biocontrol agents against bark beetle pests. In this sense, *Pseudomonas* strains belonging to well-known entomopathogenic species could be effective agents to be integrated in pest management strategies.

In this sense, field experiments analyzing the efficiency of these bacteria as biocontrol agents and the comparison of their use with other already applied methods to control this pest or even their inclusion in integrated pest management strategies should be addressed.

## 7. Conclusions and Future Perspectives

As occurs in many other environments, strains belonging to the genus *Pseudomonas* seem to have adapted to live in association with bark beetles, since they are present in most of the reports on the bacterial biodiversity associated with bark beetles. *Pseudomonas* strains are widespread among many different species of beetles from the *Scolytinae* subfamily, dispersed worldwide in different tree host species.

According to the available literature, many of these bacteria seem to be able to benefit their insect hosts by providing them with nutrients, detoxifying their microenvironment and protecting them from entomopathogens or antagonistic microbes. These reports support a predicted relevance of this genus in the bark beetles’ life cycle.

However, many of the predicted functions of *Pseudomonas* bacteria in the bark beetle holobiont are based on in vitro and in silico assays; thus, further experiments should be performed to prove whether these predictions occur in nature.

Current and future knowledge on the *Pseudomonas*–bark beetle interaction can be very useful, not only because of the inherent value of the knowledge in basic and environmental sciences, but also to aid finding the needed solutions to overcome bark beetle forest pests (either through the application of pathogenic *Pseudomonas* such as Biocontrol Agents (BCAs), or by targeting the elimination of beneficial *Pseudomonas* providing fitness to their host, weaking the beetle as the base for other biocontrol strategies).

Hence, this review aims to serve as a motivation for the development of deeper analyses in this research field to test the above--mentioned hypothesis on the role of *Pseudomonas* within the bark beetle holobiont. For instance, in vivo tests using gnotobiotic insects inoculated with the pseudomonad isolates could provide some insights into the influence of each organism to its counterpart and the application of *omics* techniques such as transcriptomics, metabolomics, and proteomics. Experiments with isotope labelled molecules might aid in the description of the molecular dialogue between both organisms.

It is also important to point out that despite the extensive literature focus on the benefits of the *Pseudomonas*-bark beetle association for the host, there is a knowledge gap on the benefits for the bacterial (*Pseudomonas*) counterpart of the bark beetle holobiont; the description of a mutual benefit would be important to define the existence of a possible mutualistic symbiosis between the macro- and the microsymbiont.

## Figures and Tables

**Table 1:** Summary of research studies in which *Pseudomonas* have been found in bark beetles including the host tree of the beetle, the sample type or body part of the beetle from where *Pseudomonas* strains have been isolated or detected, the life stage of the beetle, the method of detection (MD) of *Pseudomonas* and likely roles proposed for *Pseudomonas* strains, if any. G = gut; Ga = gallery; F = Frass; B = entire beetle crushed; P = phloem; OS = oral secretion; S = surface of the beetle; L = larvae; Pu = pupae; TA = teneral adult; PA = pre-emerged adults; A = adults; CIA= culture independent method analyses based on amplicon sequencing; CIS = culture independent method analyses based on shotgun sequencing; CD = culture dependent method.

Beetle (Tree-Host)	Sample	Life Stage	MD	Functions of *Pseudomonas*	Reference
*Dendroctonus adjunctus* (*Pinus hartwegii*), *D. approximatus* (*P. teocote*), *D. jeffreyi* (*P. jeffreyi*), *D. mesoamericanus* (*P. teocote*), *D. mexicanus* (*P. patula*), *D. parallelocollis* (*P. hartwegii*), *D. ponderosae* (*P. strobiformis*), *D. pseudotsugae* (*Pseudotsuga menziesii var. glauca*), *D. rhizophagus* (*P. arizonica*), *D. valens* (*P. leiophylla*), *D. vitei* (*P. pseudostrobus*)	G	-	CIA	-	[32]
*D. rhizophagus* (*P. arizonica*)	G	L, Pu, TA, PA, EA	CIA	-	[34]
*D. simplex* (*Larix* × *eurolepis*)	Cuticle, Ga	A	CIA	-	[27]
*D. valens* (*P. resinosa*)	Ga	-	CIA	-	[27]
*D. valens* (*P. tabuliformis*)	G	A	CIA	Prediction of metabolic activities using the PICRUSt method.	[35]
*Xyleborinus saxeseni*, *D. ponderosae*, *D. frontalis*	B, Ga	L, A	CIACIS	They found that Pseudomonas genome bins reconstructed from different insects are very similar (based on Average Nucleotide Identity (ANI) comparison).	[36]
*Xyleborus affinis*, *Xyleborus bispinatus*, *and Xyleborus volvulus* (artificially reared)	B	A	CIA	Prediction of metabolic activities using the PICRUSt method.	[37]
*Xyleborus affinis* (artificially reared)	B	A	CIA	Prediction of metabolic activities using the PICRUSt method.	[33]
*D. pondersoae* (*P. contorta* and *P. contorta—P. banksiana* hybrid)	B, Ga	A	CIS	Genetic potential for terpene and diterpene degradation.	[38]
*D. rhizophagus* (*P. arizonica*)	G	L, A	CD	Lipolytic, amylolytic, esterase, cellulolytic and xylanolytic activities.	[39]
*D. ponderosae* (*Pinus sp.*)	B, Ga, P	A	CD	*In vitro* degradation of monoterpenes.	[12]
*D. valens* (*P. montezumae* or *P. leiophylla*)	G	-	CD	Uricolytic activity. Ability to use uric acid as sole nitrogen, carbon and energy source.	[40]
*D. ponderosae* (*P. contorta*)	S	L, A	CD	-	[41]
*D. armandi* (*P. armandii*)	G	L	CD	Cellulolytic activity.	[42]
*D. valens* (*Pinus sp.*)	G, F	A	CD	Verbenone production, α-pinene degradation.	[43,44]
*D. micans* (*Picea orientalis*)	B	L, A	CD	-	[45]
*D. rufipennis* (*Picea sp.*), *D. ponderosae* (*P. contorta*)	OS	A	CD	-	[46]
*Anisandrus* (*=Xyleborus*) *dispar* (*Corylus sp.*)	B	L	CD	-	[47]
*D. valens* (*P. contorta and P. contorta—P. banksiana* hybrids)	B	A	CD	Ability to grow in presence of monoterpenes.	[48]
*Hylesinus fraxini* (*Fraxinus excelsior*)	B	A	CD	Cellulolytic activity.	[49]
*Cryphalus piceae* (*Abies alba*), *Ips typographus* (*Picea abies*)	B	A, L	CD	Degradation of cellullose, xylan, starch, and diverse chemical dyes.	[13]
*D. rufipennis*	OS	A	CD	Inhibit fungi associated with mouthparts of the beetle.	[25]
*D. ponderosae* (*P. contorta*)	OS	L, A	CD	Promote the growth of some beetle-associated fungi and suppress the growth of some other beetle-associated fungi.	[50]
*D. valens* (*P. tabuliformis*)	S, F, G	L	CD	Inoculation of different strains of *Pseudomonas* alleviate the antagonistic effects of fungi *L. procerum* and O*. minus* on larva growth. They show a direct inhibition of *O. minus* growth. They show that the D-pinitol and D-glucose consumption of O. minus is decreased, while the D-pinitol consumption of *L. procerum* is increased when bacteria is inoculated.	[28]
*I. sexdentatus* (*P. orientalis*)	B	A	CD	One isolate increase significatively the mortality when is inoculated into larvae.Another one do not increase significatively the mortality.	[51]
*D. valens*	-	-	CD	*Pseudomonas* volatiles decrease the growth of *O. minus* and, therefore, to increase the growth of larvae when *O. minus* is present.	[52]
*D. valens*	-	-	CD	*Pseudomonas* volatiles decrease the growth of *L. procerum* and, therefore, to increase the growth of larvae when *L. procerum* is present.	[53]
*D. ponderosae*	B, Ga	-	CD	Inoculation of different strains of *Pseudomonas* in *P. contorta* and in *P. banksiana*, reduced antagonistic effects by *Aspergillus* and *Trichoderma* resulting in more larvae and longer ovipositional and larval Ga.	[54]
*Ips acuminatus* (*P. sylvestris*), *Pityogenes bidentatus* (*P. sylvestris*)	B	A	CD	Ability to inhibit other microorganisms and genetic potential to produce antimicrobial compounds.	[29]
*Ips pini* (*P. resinosa*)	G	P, A	CD	-	[55]
*Hypothenemus hampei* (*Coffea arabica*, *Coffea arabica* var *limani*, *C. congensis* × *C. canephora*), *H. eruditus* (*Cecropia* sp.), *Scolytodes maurus* (*Cecropia* sp.)	B	-	CD CIA	In vitro and in vivo caffeine-degradation (use as sole source of C and N).	[56]
*Ips duplicatus*, *I. typographus* and *Polygraphus poligraphus* (*Picea abies*), *I. acuminatus* and *I. sexdentatus* (*Pinus sylvestris*), *I. cembrae* (*Larix decidua*).	G	A	CIA	Prediction of metabolic activities using the PICRUSt method.	[57]
*D. frontalis* (*P. taeda*)	G	A	CD CIA	-	[58]
